# BUILDING BRIDGES ACROSS GENERATIONS: THE INTERGENERATIONAL CLASSROOM

**DOI:** 10.1093/geroni/igae098.3562

**Published:** 2024-12-31

**Authors:** Jessica Hsieh, Raza Mirza, Christopher Klinger, Julianna Hill, Heather Janes, Claudia Osmond

**Affiliations:** University of Toronto, Toronto, Ontario, Canada; University of Toronto, Toronto, Ontario, Canada; University of Toronto, Toronto, Ontario, Canada; University of Toronto, Toronto, Ontario, Canada; Christie Gardens, Christie Gardens, Ontario, Canada; Christie Gardens, Christie Gardens, Ontario, Canada

## Abstract

The issue of aging demands greater attention in education systems, and intergenerational approaches help to combat ageism and improve the lives of older adults, now and in the future. Taking an intergenerational approach to contextualizing the experiences of older adults within post-secondary classrooms settings is a novel approach towards building interest in the field of aging. The University of Toronto (UofT) partnered with Christie Gardens, a retirement community and long-term care home, to launch an innovative experiential learning initiative: The Intergenerational Classroom. Half the learners were UofT undergraduate students (n=24); the other half were older adults residing at Christie Gardens (n=25). Through interactive seminar-style discussions, collaborative projects and mentorship, the course, which was held at Christie Gardens, provided a semester-long exploration on aging. To evaluate the program, pre/post-surveys were administered to all learners, and following the semester, students (n=6) and older adults (n=6) participated in focus group discussions. Outcomes of program success were identified across domains, including meaningful friendships and bonds created, increased awareness of aging issues, reduced ageist attitudes, and greater sense of community and civic engagement. Evaluations revealed that 92% had an excellent learning experience, 95% found the course intellectually stimulating, and that 100% would recommend the course to others. The Intergenerational Classroom explored aging from a viewpoint that considered the perspectives of both older and younger generations. Intergenerational approaches can help to create a brighter and more inclusive future for all generations, ensuring that individuals can grow up and grow older with dignity, rights, and opportunity.

